# Fishing for Nature's Hits: Establishment of the Zebrafish as a Model for Screening Antidiabetic Natural Products

**DOI:** 10.1155/2015/287847

**Published:** 2015-11-23

**Authors:** Nadia Tabassum, Hongmei Tai, Da-Woon Jung, Darren R. Williams

**Affiliations:** ^1^New Drug Targets Laboratory, School of Life Sciences, Gwangju Institute of Science and Technology, Gwangju 500-712, Republic of Korea; ^2^Department of Endocrinology, Yanji Hospital, Jilin 133000, China

## Abstract

Diabetes mellitus affects millions of people worldwide and significantly impacts their quality of life. Moreover, life threatening diseases, such as myocardial infarction, blindness, and renal disorders, increase the morbidity rate associated with diabetes. Various natural products from medicinal plants have shown potential as antidiabetes agents in cell-based screening systems. However, many of these potential “hits” fail in mammalian tests, due to issues such as poor pharmacokinetics and/or toxic side effects. To address this problem, the zebrafish (*Danio rerio*) model has been developed as a “bridge” to provide an experimentally convenient animal-based screening system to identify drug candidates that are active* in vivo*. In this review, we discuss the application of zebrafish to drug screening technologies for diabetes research. Specifically, the discovery of natural product-based antidiabetes compounds using zebrafish will be described. For example, it has recently been demonstrated that antidiabetic natural compounds can be identified in zebrafish using activity guided fractionation of crude plant extracts. Moreover, the development of fluorescent-tagged glucose bioprobes has allowed the screening of natural product-based modulators of glucose homeostasis in zebrafish. We hope that the discussion of these advances will illustrate the value and simplicity of establishing zebrafish-based assays for antidiabetic compounds in natural products-based laboratories.

## 1. Introduction

Over the past three decades, the prevalence of diabetes mellitus (DM) has dramatically increased in both developing and developed countries, affecting millions of adults, and this number is expected to increase to approximately 439 million adults by 2030 [1]. DM is a heterogeneous group of metabolic disorders that occurs either due to resistance of the body to respond to insulin or due to the insufficient production of insulin in the pancreas, both of which cause elevated levels of blood sugar (hyperglycemia) [2]. Prolonged hyperglycemia causes severe and potentially fatal complications, such as cardiovascular disease, nephropathy, and retinopathy [3]. Numerous antidiabetes drugs have been developed, but they cause side effects of varying severity, such as nausea, weight gain, or cardiovascular issues [4]. Since ancient times, herbal medicines have also been used to treat diabetes in different cultures (example reviews are [5, 6]). The bioactive natural products isolated from herbal medicines have been an important source of novel drugs for various diseases [7]. For example, between 1981 and 2002, almost half of the 877 small molecule New Chemical Entity (NCE) therapeutics were natural products or their synthetic derivatives [8]. Moreover, approximately 50% of all new commercially available drugs are developed from natural products [7]. Natural products are an attractive starting point for the drug discovery process, because many possess structural complexity that cannot be achieved using chemical synthesis. In addition, synthetic analogues of natural products can be developed, which possess superior safety and efficacy profiles [9, 10]. However, over the past two decades, natural products-based drug discovery by pharmaceutical companies has largely waned. This is because natural products are generally not suitable for large scale drug screening protocols, compared to synthetic compounds. Costs and technical difficulties associated with the isolation, purification, and quality control of novel natural products also preclude their use in the drug discovery industry. However, natural products-based drug discovery continues to flourish in academia. A recent example is the development of the anticancer drug ecteinascidin 743 (from the sea squirt,* Ecteinascidia turbinate*) (Figure 2) which obtained clinical approval to treat metastatic soft tissue sarcoma [10]. Therefore, the development of powerful, high-content screening assays for natural products would complement their established advantages as candidates for drug discovery.

Over the past two decades, major progress has been achieved in the development of screening technologies, such as high throughput screening (HTS) [11] and chemical synthesis strategies, such as diversity orientated synthesis [12]. However, the number of licensed compounds obtained against novel drug targets is considerably low (just two to three compounds per year). Moreover, a single pharmaceutical company can spend £100–500 million annually for investigating 30–50 targets [13] and a new compound typically requires 12 years to enter the drug market [14]. There are numerous reasons to account for this low number of new drugs, such as increasingly rigorous safety evaluations and the reduced efficiency of target-based discovery (known as Eroom's Law) [12]. Another major bottleneck in the drug discovery process is the failure of promising candidate compounds in animal tests, due to issues related to absorption, solubility, metabolic stability, distribution, and/or toxicity. These failures also arise because cell-based screening systems cannot recapitulate the physiological interactions that are crucial in the evolution of some disorders, such as metabolic diseases [15]. Another significant reason for the rejection of candidate drugs is that discovery methods rely mainly on the existence of identifiable and “screenable” targets [16]. This late-stage attrition of drugs in development is highly costly and time consuming for pharmaceutical companies. These drawbacks of target driven drug discovery have induced researchers to screen compounds, including natural products, in whole animal systems to identify new targets and ultimately successful drugs. In the next section of this review, we compare conventional screening systems with animal-based approaches and introduce the zebrafish as an ideal model system for* in vivo *drug discovery applications.

## 2. Development of Animal-Based Screening Systems for Drug Discovery

The mouse (*Mus musculus*) is the most widely used animal model in biomedical research and can be considered as the “work horse” model organism [28]. It possesses numerous advantages for research purposes, such as sharing 90% genome homology with humans, in addition to many genetic, physiological, and organ anatomical similarities. Another important advantage is that genetic manipulation in the mouse can be used to model the action of potential drugs, via the knockdown of candidate target genes. Thus, the mouse is an invaluable model for the discovery of new targets for therapeutic interventions [29]. However, from the perspective of drug screening, the mouse has significant disadvantages restricting its use in the pharmaceutical field. The major problem is the expense involved in maintaining large mouse colonies for compound screening. There are also biological issues. For example, transgenic animals that are used to model disease may not be suitable for screening, because of the potential initiation of compensatory gene expression mechanisms during development. This could be problematic for discovering compounds that should be active in humans [16]. Consequently, alternative animal-based platforms for screening have been investigated. Two prominent examples are invertebrate models: the fruit fly,* Drosophila melanogaster*, and the roundworm,* Caenorhabditis elegans *[30, 31]. Despite offering certain advantages, such as applicability to HTS and the preservation of some physiological contexts that are similar to humans, these invertebrate models have not become widely used for drug screening. This is because these invertebrates have markedly reduced genetic homology with humans compared to the mouse. In addition, some of the major organ systems present in humans, such as respiratory and intravascular circulatory systems, are not present in invertebrates [32]. Another issue is the thick cuticle, which affects drug penetration and complicates the interpretation of screening data (this is especially relevant for* C. elegans*) [33, 34]. Thus, the clinical potential of novel compounds discovered in invertebrates may not be sufficient to justify screening in these animal systems.

## 3. Zebrafish: Swimming into Place in the Drug Discovery Field

**Figure 1:**
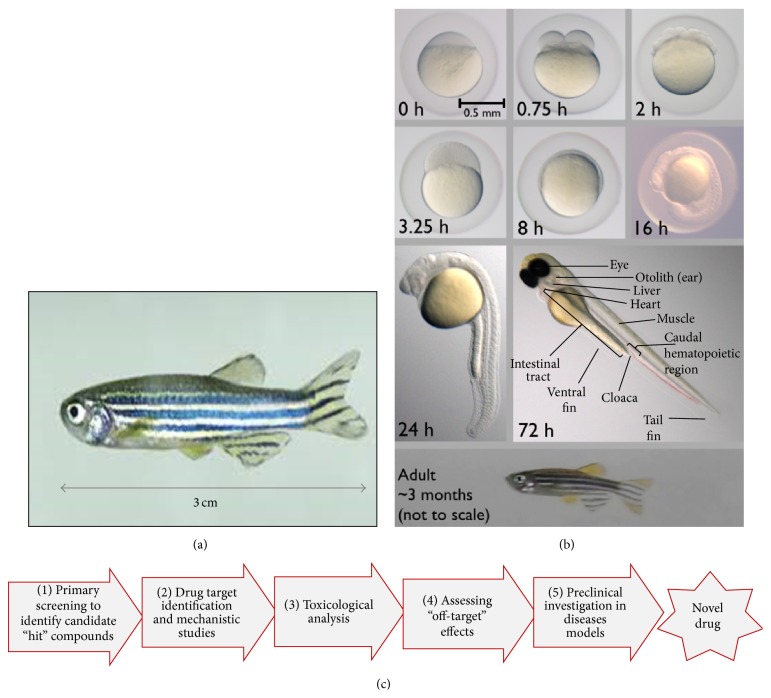
(a) An adult zebrafish. (b) Embryonic development of zebrafish is rapid, with the major organ systems, such as nervous, cardiovascular, and digestive tissues, being formed within 36 hours of fertilization [35] (image adapted from Wikimedia and used under the Creative Commons Attribution-Share Alike 4.0 International license). (c) The zebrafish model can facilitate multiple steps of the drug discovery process.

**Figure 2:**
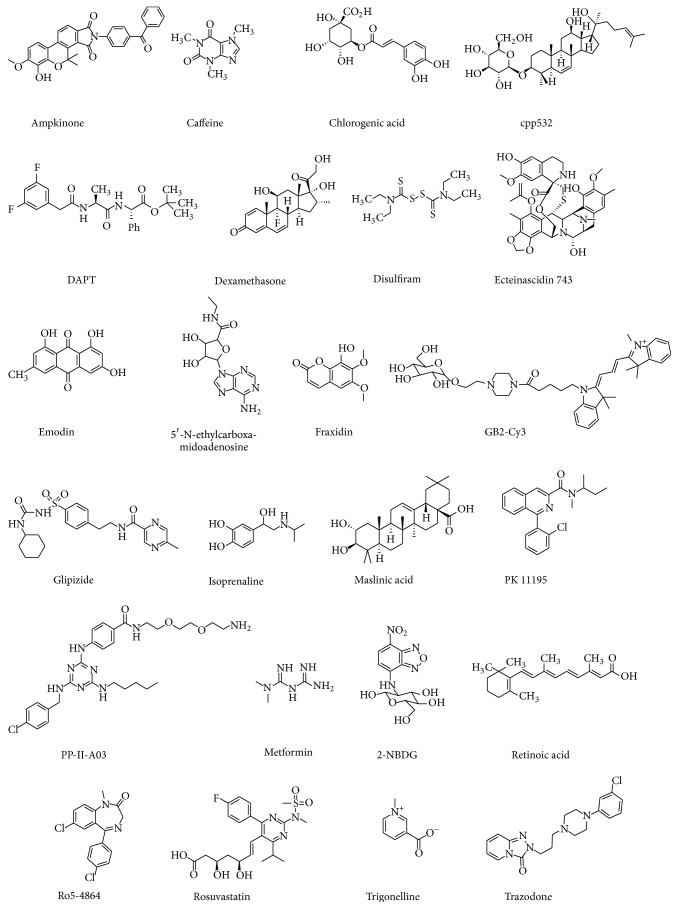
Chemical structures of compounds discussed in this review.

These above-described limitations on mammalian and invertebrate models for compound screening provided impetus to develop alternative animal model systems for studying drug responses. Consequently, the zebrafish (*Danio rerio*) has risen to prominence. The zebrafish is a small fresh water teleost that was developed as an animal model in the 1980s for the study of developmental biology and embryogenesis [36, 37]. A set of landmark studies, published together in a single issue of the journal,* Development*, (1996) helped to establish this animal model for characterizing vertebrate developmental pathways. Further studies and the production of gene targeting techniques, such as anitisense morpholinos and the generation of transgenic fish, enabled zebrafish to become an attractive model for studying human disease [38, 39]. Critically, the logistical advantages of using zebrafish have also convinced the research community to adopt this animal model. These advantages are listed as follows:

1. In contrast to rodents, zebrafish embryos are optically transparent and zebrafish development is external, which permits direct observation of embryonic organ systems under a wide variety of laboratory conditions [40]. For its size, zebrafish embryos are relatively large, which assists their handling in the laboratory.
2. Embryonic development is very rapid compared to mammals. The entire body plan is established by 24 hours after fertilization (hpf) and most major organ systems, such as the digestive tract and the cardiovascular system, are developed 2 days after fertilization (dpf). Complete embryogenesis (hatching) occurs by 72 hpf. Therefore, several different biological processes and/or disease mechanisms can be analyzed during these early developmental stages (Figure 1) [41].
3. The small size of the zebrafish embryo (5 mm at 7 dpf), relatively low cost for maintenance compared to rodents, and high fecundity (a single female can lay up to 200 eggs per week) make screening possible in 96- or 384-well microtiter plates [42, 43].
4. It is now established that drugs against certain organs or tissues that differ or are absent in humans can also be discovered or tested in zebrafish. For example, the drug, rosuvastatin (Figure 2), used to treat prostate cancer, was identified by zebrafish chemical screening even though male zebrafish do not possess a developed prostate gland, only-prostate-like cells [44].
5. In contrast to insect models for drug screening, zebrafish are vertebrates and possess higher genetic homology with humans (approximately 80%, compared to the fruit fly,* Drosophila melanogaster* (~60%), and the roundworm,* Caenorhabditis elegans* (~36%)) [13, 45, 46].

All of the characteristics listed above make zebrafish an ideal model system for drug discovery efforts (Figure 1). A comparison of the advantages of zebrafish-based drug testing approaches in relation to invertebrates and mammals, in the context of diabetes research, is shown in Table 1. It should also be noted that another prominent small teleost fish model has been developed: the medaka (Japanese rice fish;* Oryzias latipes*) [47, 48]. Medaka possesses certain advantages for laboratory use compared to zebrafish, such as a smaller genome and more rapid development time. However, zebrafish is the more established fish model and have been shown to possess some significant physiological differences compared to medaka, such as greater cardiac regeneration after injury [49]. The potential of zebrafish for diabetes-related drug screening approaches have been realized over the past decade, with multiple strategies developed, such as visual assessment of glucose uptake or quantitative analysis of glucose homeostasis using the microplate format. These developments and their application for natural products-based screening of antidiabetic compounds are described in the next section of this review.

### 3.1. The Development of Zebrafish-Based Screening for Antidiabetes Natural Products

Interestingly, the first use of zebrafish larvae to screen natural products and synthetic compounds for modulators of cell physiology was reported over fifty years ago [50]. Surprisingly, this research was largely ignored until the 1990s. In this decade, combinatorial synthesis of small molecule libraries was developed, allowing the production of compound libraries comprising thousands of small molecules [51]. This progress necessitated the requirement for rapid and efficient screening systems for bioactive compound discovery. As described in the previous section of this review, the pharmaceutical research community realized that an animal-based screening system with the potential for high throughput screening/validation could greatly assist drug discovery. Consequently, the zebrafish model was developed for compound screening based on numerous experimental “readouts” such as developmental phenotypes [24], pigmentation modulation [52–54], tissue regeneration modulation [55], and radiation sensitization [56]. The development of zebrafish-based antidiabetes compound screening is based upon the discovery of marked similarities in glucose homeostasis with mammals. These similarities, which justify the use of zebrafish to screen for antidiabetic compounds, are outlined below.

### 3.2. Zebrafish Can Be Used to Model Pancreatic Beta Cell Neogenesis

Diabetes type I is characterized by the destruction of insulin producing beta cells in the pancreatic islets, producing insulin deficiency and resultant hyperglycemia. Insulin secretion from beta cells is induced by increases in blood glucose level after meal times (postprandial). Dysregulated hyperglycemia causes deleterious effects on many organs and tissues, such as the heart, kidney, and retina [2]. Interestingly, under certain physiological conditions, such as increased metabolic demand, *β* cell mass can increase via three mechanisms: (i) differentiation of resident precursor cells, (ii) transdifferentiation of other pancreatic cell types, and (iii) *β* cell proliferation [57]. Thus, inducing *β* cell neogenesis in type 1 diabetic patients could be a promising therapeutic strategy. Moreover, inducing *β* cell transdifferentiation from other cell types could provide a cell source for transplantation into diabetic patients [58, 59].

Traditionally, the mouse has been used as a model to study the restoration of *β* cell mass. However, recent studies in zebrafish have shown its potential as a model of mammalian *β* cell regeneration. In addition, pancreas development and regeneration can be studied in transparent zebrafish embryos [60].

Despite the small size of this tropical fish, its pancreatic structure is highly similar to mammals and is also comprised of two types of glandular tissues: exocrine and endocrine. Many pancreatic developmental genes and hormones that regulate glucose metabolism in zebrafish, such as insulin and glucagon, resemble those of mammals [61, 62]. For example, the well-characterized early marker of pancreatic development,* pdx-1* (pancreatic and duodenal homeobox-1), is conserved between mammals and zebrafish [63]. Both zebrafish and mammalian embryos share the positive regulatory relationship between* hedgehog* and* pdx-1* in pancreas precursor cell specification [64]. In a related study, it was shown that perturbation of pancreatic genes in the zebrafish produced phenotypes that closely resembled the equivalent human disease. For example, mutations of the nuclear protein* vhnf1* (*tcf2*) are associated with maturity-onset diabetes of the young, type V (MODY5).* Vhnf1 *mutant zebrafish embryos displayed developmental defects in the pancreas and the liver, along with the formation of kidney cysts, which are also found in MODY5 patients [65, 66]. Interestingly, the blood glucose concentration of adult zebrafish is close to the human physiological level (50–75 mg/dL and 100 mg/dL, resp.) [3, 67].

These similarities between mammalian and zebrafish beta cells provided research impetus to develop compound screening systems for *β* cell neogenesis. For example, transgenic fish expressing fluorescent-tagged* nfsB* (dihydropteridine reductase; expressed in the embryonic pancreas) were generated to allow visualization of drug-dependent pancreatic cell ablation. The potential of this system for image-based compound screening was validated using the prodrug, metronidazole, which is known to be cytotoxic for beta cells [68]. Recently, Rovira et al. utilized a transgenic zebrafish model to carry out screening of the Johns Hopkins Drug Library (consisting of around 1500 FDA and foreign approved drugs) for compounds that promote *β* cell differentiation. Their moderate-throughput screening system used multiwell plates to permit visualization of pancreatic cells in living larvae and led to identification of three FDA approved drugs that induce significant *β* cell differentiation: (1) disulfiram, (2) DAPT (N-[N-(3,5-difluorophenacetyl)-L-alanyl]-S-phenylglycine t-butyl ester), and (3) the fungal-derived natural product, mycophenolic acid (Figure 2) [69]. In a related study, Andersson et al. used the zebrafish screening platform to discover compounds that induce *β* cell regeneration [70]. A library of approximately 7000 compounds (including FDA approved drugs and natural compounds) was screened in a transgenic zebrafish model showing *β* cell specific expression of nitroreductase [Tg(ins:CFP-NTR)], which converts metronidazole (MTZ) into a cytotoxic product that specifically kills beta cells. After the removal of tested compounds, larvae showed reconstitution of the beta cell mass, which could be quantified by microscopy. The most promising enhancer of *β* cell regeneration was found to be NECA (5′-N-ethylcarboxamidoadenosine, an adenosine analogue). NECA was further tested in a mouse model of diabetes, which validated its ability to induce *β* cell regeneration in mammals.

**Figure 3:**
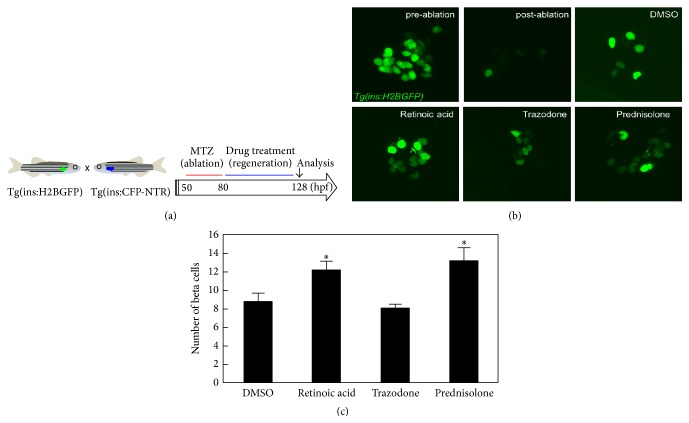
(a) Schematic diagram for zebrafish-based screening of compounds that promote beta cell regeneration. Transgenic fish expressing GFP in the beta cells (ins:H2BGFP) were bred with transgenic fish that express nitroreductase specifically in their beta cells Tg(ins:CFP-NTR). Beta cells were ablated using MTZ treatment from 50 to 80 hpf. At 80 hpf, Tg(ins:H2BGFP); Tg(ins:CFP-NTR) larvae were treated with the compounds for 48 h. The numbers of Tg(ins:H2BGFP) + beta cells were counted at 128 hpf. (b) Microscopic images of Tg(ins:H2BGFP) + beta cells in 128 hpf larvae treated with 1 *μ*M retinoic acid, 10 *μ*M trazodone, or 10 *μ*M prednisolone dissolved in 1% DMSO. (c) Quantification of beta cell regeneration per larva at 128 hpf, following treatment with hit compounds from 80 to 128 hpf. Error bars represent SEM. ^*∗*^ *P* < 0.05 compared to DMSO treated controls. Image reproduced from [71], under the Creative Commons Attribution (CC BY) license.

Until recently, drug discovery for compounds that induce *β* cell proliferation did not attract significant attention from pharmaceutical companies, due to the poorly understood signaling pathways regulating this process. Recently, a zebrafish-based screening model has demonstrated its utility for screening to identify inducers of beta cell proliferation. Tsuji et al. developed an* in vivo* imaging approach that utilized a fluorescent ubiquitylation-based cell cycle indicator. 20 small molecules among 883 were identified as having the ability to induce beta cell proliferation in zebrafish. Among these, retinoic acid (the metabolite of vitamin A) and the antidepressant, trazodone (Figure 2), have already been shown to increase mammalian beta cell proliferation indicating that zebrafish screening can detect bioactive molecules that also function in their mammalian counterparts (Figure 3) [71, 72]. Of note, it has also been shown that various active components from coffee (caffeine, trigonelline, and chlorogenic acid) can also induce beta cell regeneration in alloxan-treated zebrafish [73]. In this study, beta cells were visualized using Ins:GFP transgenic fish or by simply treating fish with a fluorescent-tagged glucose tracer.

Interestingly, it is now possible to model the effects of insulin resistance on beta cell numbers using the zebrafish system. Transgenic fish were generated that overexpressed adominant-negative version of the insulin-like growth factor-1 receptor [6]. Young fish show normal glucose tolerance, because they respond to insulin resistance by inducing beta cell proliferation. However, in older fish this response became less effective and beta cell numbers decreased, which produced insulin resistance. Thus, it can be envisaged that antidiabetics drug candidates that target insulin resistance can be assayed using these transgenic zebrafish.

### 3.3. Zebrafish as a Model Animal for the Quantitative Analysis of Glucose Homeostasis

For effective diabetes drug discovery research using zebrafish, it would be desirable to study blood glucose regulation in this model and correlate its relationship with the human metabolic system. Although the small size of zebrafish embryos precludes the collection of blood samples for measuring glucose level, this is possible in adult zebrafish. For example, methods for the microsampling of whole blood and plasma have been developed for measuring blood glucose in fasting and refed zebrafish [67]. To measure glucose levels in zebrafish embryos, which are becoming suitable for high throughput screening [74, 75], Jurczyk et al. developed a fluorescent, dual enzyme assay to detect free glucose in the embryos [76]. This technology demonstrated that zebrafish pancreatic islets produce a regulatory glucose system at an early developmental stage (48 hpf). In addition, targeting zebrafish* pdx-1* gene expression during embryogenesis produced islet hypoplasia and persistent hyperglycemia. This result correlates with previous studies reporting that impaired* pdx-1* activity causes defective pancreas development in humans [77, 78].

The regulatory mechanisms controlling blood glucose levels in zebrafish also share many similarities with mammals. Elo et al. tested three FDA approved antidiabetic drugs: glipizide, metformin, and rosiglitazone (Figure 2) in zebrafish [79]. 96 hpf larvae were exposed to cAMP and dexamethasone to activate zebrafish phosphoenolpyruvate carboxykinase (zfPEPCK), which regulates blood glucose levels via gluconeogenesis in the liver and kidneys of mammals [80, 81]. Well-known antidiabetes drugs, such as metformin, downregulate PEPCK expression and this enzyme is used as a readout in mammalian cell culture models to check the efficacy of antidiabetic compounds [26, 82]. Glipizide, metformin, and rosiglitazone all successfully inhibited cAMP/dexamethasone activation of zfPEPCK expression, even after treatment with relatively small doses, such as 1 *μ*M for rosiglitazone. These findings were significant for zebrafish-based antidiabetes drug discovery, because it indicated that hypoglycemia-inducing drugs, which function via PEPCK inhibition, could be detected using zebrafish larvae, which are amenable for 96-well plate format screening [79]. Subsequent to this study that was based on measuring zfPEPCK expression via quantitative RT-PCR, Gut et al. engineered transgenic zebrafish with a luciferase luminescent PEPCK reporter to utilize larvae in a HTS platform [22]. To validate their screening system, two known modulators of blood glucose level in humans were tested: metformin, which induces hypoglycemia, and isoprenaline, which induces hyperglycemia (Figure 2) [83–85]. Isoprenaline and metformin strongly induced or reduced PEPCK reporter expression in zebrafish larvae, respectively. Glucose levels in the larvae were also reduced by metformin and this reduction could be overcome by cotreatment with isoprenaline. 2400 compounds were screened (a collection of natural compounds, FDA approved drugs, and other bioactive chemicals). 60 compounds were identified as modulators of PEPCK expression. Interestingly, two of the most prominent hit compounds, the translocator protein (TSPO) ligands PK 11195 and Ro5-4864, decreased glucose levels in the larvae while producing increased PEPCK expression. In a mammalian model of diet-induced obesity, these compounds reduced blood glucose level intolerance and inhibited the development of hepatosteatosis (fatty liver disease). Thus, compounds that modulate PEPCK expression in larval zebrafish are drug candidates for treating metabolic diseases, such as diabetes, in mammals.

**Figure 4:**
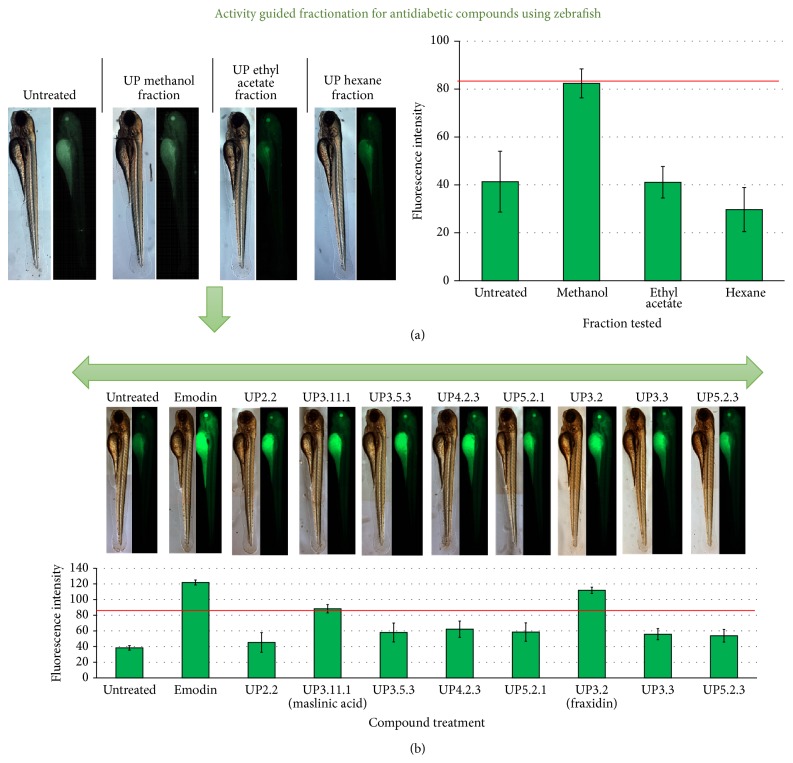
Activity guided fractionation of the inner shell of the Japanese chestnut tree,* Castanea crenata*, using the fluorescent probe 2-NBDG in zebrafish larvae. (a) The methanol fraction produced significant glucose uptake in zebrafish compared to the hexane or ethyl acetate fraction. The red line on the graph indicates the threshold for selecting a “hit” drug (i.e., 2-NBDG uptake value for the zebrafish eye should show a ≥100% increase compared to that of the untreated larvae). (b) The methanol fraction was purified to isolate eight compounds: UP2.2 (scopoletin 4), UP3.11.1 (maslinic acid), UP3.5.3 (fragransin), UP4.2.3 (4-ketopentanoic acid), UP5.2.1 (4-hydroxy-5-methoxycinnamic acid), UP3.2 (fraxidin), UP3.3 (6,7,8-trimethoxycoumarin), and UP5.2.3 (3,4,5-trimethoxycinnamic acid). These compounds were tested for glucose uptake in the zebrafish (10 *μ*g/mL dose for 1 h) and compared with emodin (a known inducer of glucose uptake). Fraxidin and maslinic acid were identified as hit compounds for inducing glucose uptake (figure modified from [26]).

An ideal approach for detecting compounds that affect glucose homeostasis in zebrafish would be to directly visualize glucose flux* in vivo*. Fluorescent-tagged glucose bioprobes have been used to visualize glucose uptake in cells (reviewed in [86]). Lee et al. utilized these probes to develop a screening system for measuring glucose flux in zebrafish larvae [26].This screening system is relatively simple, because it is applicable for wild type zebrafish (transgenic fish may be subject to quarantine controls) and glucose flux is measured using the commercially available probe, 2-(N-(7-nitrobenz-2-oxa-1,3-diazol-4-yl)amino)-2-deoxyglucose (2-NBDG; Figure 2). Glucose flux in 72 hpf zebrafish larvae could be assessed by fluorescent microscope analysis of the zebrafish eye, which expresses relatively high levels of glucose transporter (GLUT) proteins [87]. Their screening system was validated using the natural product purgative resin, emodin (6-methyl-1,3,8-trihydroxyanthraquinone; Figure 2), which is a known inducer of glucose uptake [88]. Antidiabetic compounds that induce glucose uptake could also be quantified by lysing the larvae and measuring 2-NBDG fluorescence in a microplate reader. Interestingly, the applicability of this screening system for natural products-based research was demonstrated using activity guided fractionation of compounds from the inner shell from the Japanese chestnut tree (*Castanea crenata*) (shown in Figure 4). The strongest performing compound was identified as fraxidin, which had no previously reported antidiabetic activity. This compound was confirmed as novel insulin mimetic with activity in mammals via testing in a mammalian adipocyte system. The known antidiabetic compound, maslinic acid (Figure 2) [89], was also identified in this study. Additionally, screening of a collection of saponin based natural products isolated from Korean ginseng (*Panax ginseng*) allowed identification of a novel antidiabetic compound, termed cpp532 (Figure 2). Visualizing glucose homeostasis via fluorescent probe uptake in the eye is the first demonstration that glucose flux in a zebrafish-based system can be monitored directly and offers a novel approach for antidiabetic drug discovery [26]. Interestingly, the 2-NBDG glucose probe has also shown applicability for visualizing glucose uptake by insulin sensitive tissues in mice [27, 86].

**Figure 5:**
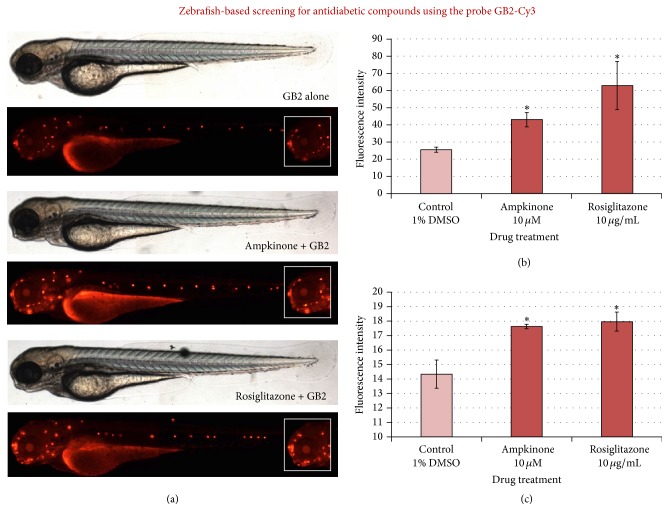
The fluorescent probe GB2-Cy3 can be used to test candidate antidiabetic compounds in zebrafish. (a) The known antidiabetic compounds ampkinone (10 *μ*M) and rosiglitazone (10 *μ*g/mL) increased probe uptake in larval zebrafish. From a screening perspective, probe uptake can be readily quantified by fluorescent microplate reader analysis of lysed larvae for GB2-Cy3 uptake (b) or image-based quantification of fluorescent signal from the zebrafish eye (^*∗*^ = *p* < 0.05 compared to the control group) (figure reproduced with permission of the Royal Society of Chemistry from [92]).

Unfortunately, the widely available 2-NBDG glucose probe does have certain disadvantages that may restrict its use for visualizing glucose homeostasis* in vivo*. For example, 2-NBDG requires a high treatment concentration (600 *μ*M for zebrafish), suffers from rapid photobleaching, and has relatively low sensitivity compared to recently developed glucose probes which possess stronger fluorophores, such as Cy3 [90]. To further optimize the zebrafish larvae-based screening system for antidiabetes drug discovery, the 2-NBDG probe was replaced with GB2-Cy3 (Figure 2) [91, 92]. Cell-based analyses had previously shown the superior imaging properties of GB2-Cy3 compared to 2-NBDG [93]. In zebrafish larvae, it was shown that GB2-Cy3 is approximately tenfold more sensitive for monitoring glucose flux and could be used at a treatment concentration as low as 5 *μ*M (120 times lower than 2-NBDG) [92]. The sensitivity of GB2-Cy3 fluorescence to known modulators of glucose homeostasis was demonstrated using the natural product, emodin (Figure 2). Additionally, two other known antidiabetic drugs were tested to validate the GB2-Cy3 probe in this zebrafish system: ampkinone and rosiglitazone (Figure 5) [94]. These results indicate that zebrafish larvae-based* in vivo* screening for antidiabetic natural products using the probe GB2-Cy3 would be more experimentally robust compared to screening based on the 2-NBDG probe. However, it should be noted that test screening, such as the activity guided fractionation approach described for 2-NBDG (Figure 4), was not attempted for the GB2-Cy3 probe. In addition, unlike 2-NBDG, GB2-Cy3 is not commercially available at this time, which restricts the suitability of this probe for use by the natural products research community.

### 3.4. Candidate Antidiabetic Compound Validation in Zebrafish-Based Models of Diabetic Complications

As mentioned in the introduction of this review, the zebrafish offers numerous logistical and technical advantages for the assessment of new drug candidates, prior to preclinical analysis in rodent-based models. In the context of antidiabetes drug discovery, zebrafish that manifest the secondary complications of diabetes, such as kidney disease or retinopathy, would be a valuable resource for simple, initial validation of “hit” compounds identified by screening. In humans, diabetes produces numerous debilitating complications, such as neuropathy, nephropathy, retinopathy, cardiovascular diseases, peripheral artery disease, stroke, periodontal disease, and increased susceptibility to opportunist infections [95, 96]. A number of mammalian models of type 2 diabetes present secondary complications that are observed in humans (reviewed in [97, 98]). Interestingly, zebrafish models of diabetic complications have been developed, which allow experimentally convenient assessment of novel antidiabetic compounds in a vertebrate system.

A significant advantage of using zebrafish for diabetes research is that hyperglycemia can be induced by simply adding glucose to the fish water (e.g., [99]). In contrast, rodent models of diabetes typically require the injection of the toxic glucose analogues, streptozotocin, or alloxan, which preferentially kill pancreatic *β* cells. However, these analogues also produce significant side effects. For example streptozotocin is tumorigenic in the kidney, lung, and liver and alloxan produces liver and kidney necrosis as a byproduct of its metabolism [100].

It has been shown that diabetic retinopathy can be modelled in zebrafish [18]. Over a 4-week period, the fish were immersed in a 2% or 0% glucose solution, alternating between the two solutions every 24 h, which was shown to induced hyperglycemia spikes (0 versus 2% glucose). After 4 weeks, the eyes were dissected and assessed by microscopic examination. Fish exposed to high glucose water presented decreased thickness of the retinal inner plexiform layer (IPL) and inner nuclear layer (INL), which is also observed in diabetes patients. By comparison, these retinal layers in diabetic rats rendered by streptozotocin treatment showed less thinning compared to hyperglycemia zebrafish [3]. A subsequent study based on the same method to induce hyperglycemia reported that thickened, dilated blood vessels were present in the central region of the retina, which resembles the pathophysiology observed in human patients [101, 102]. During the proliferative stage of diabetic retinopathy, the cytokine vascular endothelial growth factor (VEGF) is upregulated and induces blood vessel cell proliferation (angiogenesis). Upregulated VEGF expression was also observed in the diabetic zebrafish. This provides a relative straightforward model system to test novel antidiabetic compounds for preventative effects on the progression of retinopathy, because VEGF has been identified as a promising drug target for this complication [103].

Cardiovascular issues, such as coronary heart disease and stroke, are major complications of diabetes [104]. Exposure of 6 hpf zebrafish embryos to 0.5% glucose water until 24 hpf produced defective cardiac development and altered expression of major cardiac markers. Therefore, the potential for novel antidiabetic compounds to protect against cardiovascular complications could be tested in this zebrafish system [105]. Kidney nephropathy is another major complication of diabetes and is a major cause of dialysis in developed countries [106]. Prolonged hyperglycemia results in diffuse scarring and thickening of the glomerular basement membrane (GBM) in the kidney. This diabetic complication can also be modelled in zebrafish [3]. Microscopic analysis revealed a significant increase in zebrafish kidney GBM thickness 3 weeks after the onset of diabetes. Interestingly, in this study, diabetes was induced in the fish by intraperitoneal injection of streptozotocin (as an alternative route, streptozotocin was also be injected into the caudal fin). In addition, disruption of the zebrafish ortholog of solute carrier family 12 member 3 (a sodium/chloride transporter in kidney that is linked to diabetic nephropathy (DN) in humans [107]) produces histopathological changes in the kidney that resemble human DN [108]. Thus, the potential protective effects of novel compounds on DN can also be readily assessed in the zebrafish system.

Diabetes produces defects in wound healing, which is linked to hyperglycemia-induced negative regulation of insulin-responsive growth factors, such as insulin-like growth factor-1 (IGF-1) [109, 110]. This leads to complications, such as diabetic foot ulcers, which are a leading cause of amputations affecting 15% of all diabetes patients [111]. Of note, this complication can also be modeled in zebrafish and provides an opportunity for compound screening to identify enhancers of diabetic wound healing [3]. Healthy zebrafish readily regenerate different tissue types after amputation/resection, such as fins, spinal cord, and even portions of the ventricle, brain, or retina [112]. The caudal fin of zebrafish is a particularly attractive model for studying the molecular mechanisms regulating regeneration due to the fin's relatively simple structure, which is unnecessary for survival and undergoes rapid regeneration [113]. Amputation of the caudal fin in diabetic zebrafish resulted in reduced healing/regeneration compared to fish with normoglycemia, which could be imaged readily using light microscopy. Thus, the zebrafish fin amputation diabetic model can be used to determine the signals and mechanisms regulating regeneration in the context of diabetes [3].

Overall, the zebrafish has been used successfully to model numerous diabetic complications observed in humans. This provides an experimentally attractive model system for the rapidly confirming the therapeutic effect(s) of novel antidiabetic compounds identified by zebrafish-based screening.

## 4. Conclusion

Diabetes is a serious threat to human health and numerous drug treatments have been developed for this disease. However, none of the currently approved drugs can completely cure diabetes and they are associated with side effects, such as gastrointestinal problems for the commonly prescribed drug, metformin [114]. Late stage type II diabetes can be effectively treated by a combination therapy of oral hypoglycemic drugs plus insulin [115]. However, insulin requires subcutaneous injection for delivery (inhalable forms of insulin have been developed, but they are unlikely to be cost-effective [116]). Therefore, there is an urgent research need to develop new, more effective antidiabetic drugs that produce fewer side effects. In this review, we described the development of diabetes relevant assays using the experimentally convenient zebrafish model system and discuss the advantages of this model for natural products research. Within the past 25 years, the popularity of natural products research has diminished in the drug discovery field, because of major advances in the molecular biology field and the establishment of combinatorial chemistry. These advances provided the technology to design compounds that target specific drug targets. However, there is renewed interest in natural products-based drug discovery and development, which is due in part to the establishment of the “-omics” sciences, such as proteomics, genomics, and metabolomics, which allow detailed characterization of the effects of natural compounds on global gene expression patterns and complete signaling pathway analysis (these advances are discussed in more detail in [117]). As described in this review, the zebrafish is now established as a powerful, validated screening system for drug discovery prior to preclinical testing in mammals. Although the application of this system to antidiabetes drug discovery is relatively recent, compared to other research fields (such as cancer therapeutics), it is now known that zebrafish and humans share significant, overlapping biological mechanisms to regulate glucose homeostasis. These mechanisms can be analyzed in fish larvae in a 96-well plate format that facilitates drug discovery screening [79]. Remarkably, the zebrafish system can be used to test compounds that show high potential for antidiabetes drug development, such as novel modulators of pancreatic *β* cell regeneration, which are destroyed in type 1 diabetes. The great advantage of using zebrafish-based screening for antidiabetic drug candidates is that it only requires wild-type fish, which can be purchased from a pet store [26]. Just two or three fish tanks are needed to set up a small scale zebrafish facility. Additional equipment, such as breeding chambers and a culturing cylinder for* Artemia* (a preferred food source for zebrafish), is also readily available at low cost or can even be “home-built” [118]. Thus, a basic zebrafish setup can be housed on a single bench in the laboratory. From the standpoint of natural products research, a zebrafish based assay system can be incorporated into the laboratory using a similar amount of space as a cell culture facility, but with reduced set-up and running costs. Therefore, a natural products research laboratory with an interest in drug screening and validation could employ the zebrafish system to provide vertebrate-based assays for their compounds. Such animal-based analysis could potentially enhance the scope and impact of their research. From the perspective of diabetes drug discovery, the regulatory mechanisms of glucose homeostasis in zebrafish have been shown to possess significant homology with humans (e.g., [79]). Over the past ten years, much research progress has been achieved in establishing zebrafish as a diabetes animal model. This can be achieved relatively simply, by a single injection of streptozotocin or immersion in high glucose water [119]. Of significance, pathophysiologic aspects of diabetes can also be modelled, such as beta cell loss and epigenetic modifications that produce “metabolic memory” after the onset of hyperglycemia [68, 120, 121]. These discoveries have laid the foundation for antidiabetes drug screening using zebrafish and provide further validation that compounds discovered in zebrafish can also be effective in mammals. Therefore, zebrafish can be used to circumvent the major “bottleneck” in drug discovery, which is the failure of primary hits from cell-based screens to be effective in mammalian model systems.

Two major types of screening protocol have been developed for antidiabetic compounds in zebrafish and are validated in mammalian systems [22, 26]. One protocol is based on transgenic zebrafish larvae expressing a fluorescent PEPCK reporter gene [22] and the second is based on monitoring of glucose homeostasis using a fluorescent bioprobe [26, 92]. The advantage of the second protocol is that it employs wild-type fish and does not require prior knowledge of the drug target for screening. The advantage of the first protocol is that modulators of the well-known antidiabetes drug target PEPCK can be identified.

From the viewpoint of natural products research it has been demonstrated that activity guided fractionation of antidiabetic compounds from plant extracts can be carried out in zebrafish. In addition, a library of natural products has been screened in this zebrafish system, which produced the discovery of a novel antidiabetic drug candidate that is effective in a mammalian system [26]. The development of larval zebrafish-based screening for antidiabetic agents is a recent research advance that allows natural products scientists' access to a simple, convenient, and cheap assay for testing their novel compounds* in vivo*.

The zebrafish is gaining more interest as a tool for drug discovery because it has been demonstrated that pharmacokinetic analyses can be undertaken in this model [122, 123]. Moreover, a recent study has shown that the effect of compound glycosylation on biological activity can be assessed in the transparent zebrafish larvae [124]. This is especially relevant for natural products research, because plants generally store chemicals as glycosides, which are then activated by enzyme hydrolysis [125]. Thus, the zebrafish model can be considered as a “stand-alone” system in which antidiabetes drug screening, validation, effects on secondary diabetic complications and pharmacokinetics can be investigated. Overall, we hope that this review has raised awareness of the attributes of zebrafish-based screening and will encourage natural products researchers to use this model to screen or validate their novel compounds for antidiabetic activity.

## Figures and Tables

**Table 1:** Comparison of different animal models for screening antidiabetic compounds.

Invertebrates	Zebrafish	Rodents	Large mammals (e.g., dogs and rabbits)
Silkworms can develop diabetes mellitus when fed a glucose-rich diet [17].	They can induce DM by simple immersion in high glucose water [18].	Developed diabetes mellitus within a few days by chemical injection or after a few weeks, via feeding a high-fat diet [19].	Developed diabetes mellitus by removing their pancreas [20].
Primary screening of antidiabetic drugs is possible [21].	High throughput screening for antidiabetic compounds is possible (e.g., [22]).	High throughput screening for antidiabetic compounds is not possible.	High throughput screening for antidiabetic compounds is not possible.

Requiring less time to develop diabetes mellitus compared to some mammalian models.	They require less time for screening and less amount of test compound relative to mammalian models.	Time consuming as it may take several days to develop diabetes mellitus via feeding [23].	They take several days to develop diabetes mellitus [23].

Inexpensive to use invertebrate models and less logistical requirements compared to mammals.	Inexpensive and easier to handle compared to mammalian models.	Expensive and harder to handle due to relatively large size (compared to fish or invertebrates).	Expensive and logistical/handling problems due to large size.

Reduced ethical issues compared to mammalian models.	Both adult and larval zebrafish are suitable for screening studies.	Some ethical issues depending on country of use (e.g., secured housing required in UK or USA) [24].	Ethical issues as an experimental model.

Amenable to fluorescence-based imaging and quantification of glucose uptake [25].	Fluorescence imaging of whole organism is possible for glucose uptake analysis; diabetes-related reporter gene based screening is also possible [26].	Mouse are amenable for fluorescence tracer-based imaging of whole-body insulin sensitivity and hepatic glucose production [27].	Fluorescent-based imaging of glucose homeostasis not possible.

## Abbreviations

2-NBDG: 2-(N-(7-Nitrobenz-2-oxa-1,3-diazol-4-yl)amino)-2-deoxyglucose
cAMP: Cyclic adenosine monophosphate
DEX: Dexamethasone
DM: Diabetes mellitus
DN: Diabetic nephropathy
dpf: Days after fertilization
FDA: Food and Drug Administration
GBM: Glomerular basement membrane
Glipizide: GLIP
GLUT: Glucose transporter
Hh: Hedgehog signaling pathway
hpf: Hours after fertilization
HTS: High throughput screening
INL: Inner nuclear layer
IPL: Inner plexiform layer
Met: Metronidazole
NTR: Nitroreductase
*pdx-1*: Pancreatic and duodenal homeobox-1
PEPCK: Phosphoenolpyruvate carboxykinase
PSL: Photoreceptor layer
PTU: 1-Phenyl-2-thiourea
ROSI: Rosiglitazone
STZ: Streptozotocin
TriCA: Tricyclic antidepressants
VS: Virtual screening.
